# Facilitators and barriers for return to work among patients with post-COVID-19 condition: a qualitative interview study

**DOI:** 10.1080/02813432.2025.2525434

**Published:** 2025-07-01

**Authors:** Aleksandra Sulg, Aki Vuokko, Kirsi Kvarnström, Mikko Varonen, Antti Malmivaara, Jari Arokoski, Helena Liira

**Affiliations:** ^a^ Outpatient Clinic for Persistent Symptom Rehabilitation, University of Helsinki and Helsinki University Central Hospital, Helsinki, Finland; ^b^ Terveystalo, Occupational Health Clinic, Helsink, Finland; ^c^ Finnish Institute of Occupational Health, Helsinki, Finland; ^d^ Finnish Institute for Health and Welfare, University of Helsinki, Orton Orthopedic Hospital, Helsinki, Finland; ^e^ Department of Internal Medicine and Rehabilitation Medicine, Division of Rehabilitation Helsinki University Central Hospital and University of Helsinki, Helsinki, Finland

**Keywords:** Post-COVID-19 condition, return to work, work ability, occupational rehabilitation, occupational health

## Abstract

**Background:**

Post COVID-19 condition (PCC) can have long-lasting adverse effects, including impacts on work ability. This study explores the facilitators and barriers in the return-to-work (RTW) process.

**Design and methods:**

Conducted in spring 2023 at the Outpatient Clinic for Long-Term Effects of COVID-19, this qualitative study involved phone interviews with 32 patients with PCC, of whom 28 were included in the analysis, while four interviews served as pilots. A research doctor conducted semi-structured interviews covering work ability, RTW actions and rehabilitation experiences. The interviews were recorded, transcribed verbatim and analyzed using an inductive approach.

**Results:**

Several factors influenced work ability and the RTW process. For individual-related factors, self-guided rehabilitation, stress management, a positive attitude and high motivation supported RTW. Severe symptoms like fatigue and cognitive impairment, along with negative thoughts about them and experience of stress, hindered progress. Work-related factors included supportive employers and flexible work arrangements, while negative attitudes, skepticism about PCC and inflexible workloads were barriers. Health care-related and social factors showed that adequate emotional support and comprehensive healthcare services facilitated rehabilitation, whereas poor support, limited services and insufficient PCC understanding were obstacles. Regarding social insurance, partial sick leave supported RTW, but unmet criteria for benefits posed a barrier.

**Conclusion:**

PCC’s multifactorial nature, complicated by work ability challenges, requires a holistic approach considering individual, social and work-related factors. Effective support involves understanding patients’ experiences and fostering collaboration among healthcare providers, employers and the social security system to facilitate RTW, especially in prolonged cases.

## KEY POINTS

- The post-COVID-19 condition (PCC) can impair daily functioning and work ability, presenting complex challenges for returning to work (RTW).
- Self-guided rehabilitation and positive attitudes facilitate RTW, whereas severe symptoms, negative thoughts and stress pose barriers.
- Flexible work arrangements, emotional support and being heard by various stakeholders promote RTW, while employer skepticism and inadequate healthcare services hinder the process.
- A holistic approach, emphasizing collaboration among patients, healthcare providers and employers is crucial for staying at work and supporting RTW.

## Introduction

Post-COVID-19 condition (PCC), as defined by the World Health Organization in 2021, can affect individuals who have a history of confirmed or probable SARS-CoV-2 infection [1]. Symptoms typically emerge within three months after the onset of COVID-19 and persist for at least two months and do not have an alternative explanation [1]. PCC can negatively impact various aspects of life [2], including quality of life, mental health [3], daily functioning [4], social interactions and work ability [5]. A Danish registry-based study found that about 1.5% of the population remains absent from work six months after a confirmed COVID-19 test [6].

Research indicates that many patients with PCC struggle with work ability and returning to work (RTW) [7]. Some studies report prolonged work absences lasting months or even years [8,9] and many continue to face challenges upon RTW [10]. Lack of understanding about PCC in workplaces and among health professionals has been shown to hinder rehabilitation and the RTW process [11]. Patients hospitalized due to COVID-19 infection often experience worse RTW outcomes [5,6]. At the workplace, patients with PCC benefit from modifications to the work task and reductions in workload [9,12]. PCC shares similarities with other persistent physical symptoms (PPS), such as chronic fatigue, in its symptomatology and impact on work ability. Both conditions can lead to severe functional impairment and work disability. Many patients with chronic fatigue struggle to return to full-time work, often requiring reduced hours or less physically demanding roles [13].

In Finland, occupational health service (OHS) units serve as the primary healthcare providers for employees, covering 95% of the workforce. These units have statutory responsibilities to evaluate and support work ability, for example, in the RTW process. They collaborate closely with employers, offering expert medical insights. The Finnish social insurance system provides financial support, such as sickness benefits, to individuals unable to work for health reasons. This support is largely based on assessments by OHS physicians regarding an individual’s current work ability and the RTW ability from a medical standpoint.

Work ability involves having the necessary skills, health and qualities to manage work tasks, assuming tasks are reasonable and that the work environment is acceptable [14]. It is related to aspects of employability, work absence, productivity and the ability to RTW [15]. The positive effects of work are well-documented, with strong evidence showing that re-employment improves self-esteem, enhances general and mental health and reduces psychological distress [16]. Besides health-related factors, work ability is influenced by the work environment, social factors and various individual characteristics, including beliefs and motivation [17].

However, there is limited information on the experiences of patients regarding factors that influence work ability and RTW in non-hospitalized patients with PCC, as most research focuses on hospitalized patients with acute COVID-19 infection [5,6]. Patients with a history of intensive care during acute COVID-19 are at risk of post-intensive care syndrome (PICS) but make up only a small fraction of all PCC cases. Since they may not best reflect PCC, we find it important to study RTW in a more representative patient group. This study aims to explore the perceptions of patients with PCC about how the condition has impacted work ability and the RTW process, identifying facilitators and barriers to gain a better understanding of their needs and this complex phenomenon. Ultimately, the goal is to improve support and aid targeting in the RTW process.

## Material and methods

### Study design and sample

This qualitative interview-based study is part of a larger PCC cohort study that examines the prognosis of PCC and identifies factors related to its onset, work ability and other outcomes [18,19]. Conducted at the Outpatient Clinic for the Long-Term Effects of COVID-19 at Helsinki University Hospital (HUS), Finland, it is the only clinic in the country providing rehabilitation for PCC. Patients admitted to the clinic experienced severe and persistent symptoms following primarily mild or moderate home-treated COVID-19 infection. Referrals were mainly from primary health care, with fewer from other university hospital clinics across Finland that required more intensive rehabilitation. All patients who agreed to participate and met the inclusion criteria for PCC were prospectively recruited into the cohort, as detailed in the protocol paper [18].

Patients for this study were selected from the PCC cohort study using purposive sampling. We focused on patients who had a COVID-19 infection in 2022 to ensure a more uniform timeframe since the infection. During this period, clinic admission required a positive laboratory PCR test for COVID-19 (Omicron variant). Additional inclusion criteria were working age (18–65 years) and either currently employed or having a planned RTW. Employment status was verified when inquiring about participants’ interest in the study.

**Figure 1.**
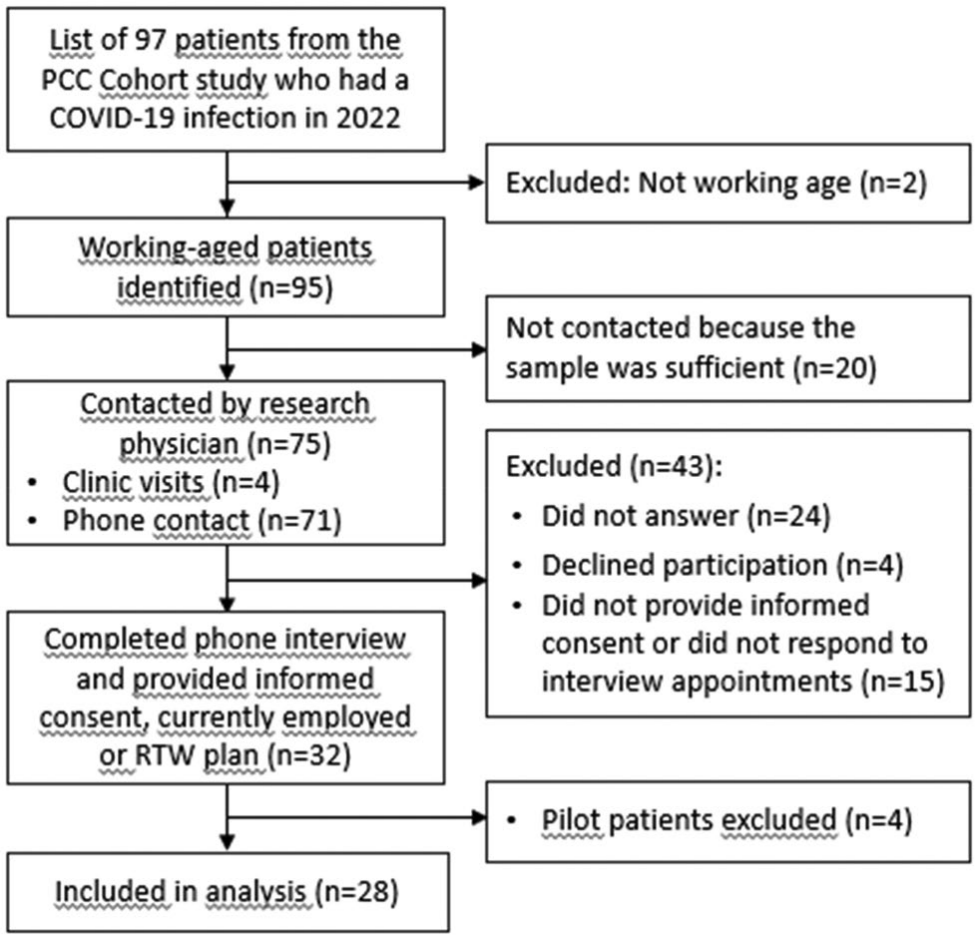
Participant flowchart. PCC: post-COVID-19 condition; RTW: return-to-work; n: numbers.

The first author (AS) received a list of 95 working-aged patients from the cohort data (Figure 1). Four patients were approached during clinic visits, and 71 were contacted by phone, starting from those with the shortest duration since clinic admission (the bottom of the list), to provide information on the study aims and inquire about their interest in participating. Of these contacts, 24 did not answer, four declined, and 15 did not provide written informed consent or did not respond to interview appointments. All participants provided written informed consent before the voluntary phone interview, with no compensation offered. AS conducted the interviews in spring 2023, engaging 32 patients in Finnish or English. Each interview lasted approximately 30 min, was audiotaped and transcribed verbatim. The first four interviews served as a pilot to test the semi-structured interview technique, question phrasing and the recording and organization process. The pilot aimed to ensure that the technique captured patients’ experiences and perspectives openly. No major changes were made through piloting, however, these pilot interviews were excluded from the final analysis to maintain a clear distinction between the piloting phase and the formal data collection process, resulting in a sample size of 28. The questionnaire required no changes after the pilot phase. Data saturation began to be observed after 15 interviews, characterized by the repetition of the same information from the patients [20].

Before the interviews, each patient visiting the outpatient clinic underwent a clinical evaluation by a physician. Additionally, prior to referral, the attending physician, whether a general or occupational health specialist, systematically excluded other potential medical conditions that could explain the symptoms. This evaluation involved assessing whether the symptoms could be linked to pre-existing comorbidities. At the clinic, patients also received treatments, including counseling for self-guided rehabilitation from a multidisciplinary team, which included physicians, physiotherapists, psychologists, social workers and nurses. Symptomatic medical treatment was provided as needed. Some interviewed patients participated in group physiotherapy sessions led by clinic physiotherapists, a parallel randomized controlled study on body-mind meditation (Amygdala-Insula retraining program, AIR) [21], or cognitive-behavioral-therapy (CBT)-based online therapies for persistent symptoms [22].

### Measures and data collection

Our data consisted of interview recordings, demographic information (including sex, age, education level, social status and occupation), physician-diagnosed diseases and self-assessment measures at baseline and during the interview. Demographic and clinical data, including a baseline survey at the first clinic visit [18], were available for all patients (*n* = 95).

We used a semi-structured interview format (Table 1) that incorporated both open-ended and closed questions. These questions covered topics, such as work ability, daily functioning, work situation, RTW supporting measures, including occupational health interventions and the workplace arrangements, as well as perceived rehabilitation experiences. The structured closed questions aimed to provide a flexible framework that encouraged broader discussions on various aspects of RTW.

**Table 1.** Overview of semi-structured interview guide on health, work ability and return to work (RTW).

Self-assessed health, daily functioning and work ability
Current health: VAS 0 (low) to 10 (high)
Daily functioning in work, social life and home: VAS 0 (not able) to 10 (excellent) [23,24]
Current work ability: VAS 0 (total disability) to 10 (best ability) [25]
Current ability to work
Work ability assessment: fully able, partially able, completely unable [27].
Reasons for reduced work: sick leave, partial sick leave, (partial) disability pension, other
Readiness and factors affecting RTW
Readiness to RTW
Discussion on factors that aid and hinder work ability and RTW
OHS and RTW measures
Availability and use of OHS for symptoms and RTW issues
Experience with health professionals: VAS 0 (not heard) to 10 (fully heard)
Experience with RTW negotiations and their usefulness: VAS 0 (not useful) to 10 (very useful)
Discussion on helpful workplace arrangements and rehabilitation strategies

*Note:* VAS: Visual Analogue Scale; OHS: occupational health service.

To enhance these discussions, structured questions utilized a Visual Analog Scale (VAS) ranging from 0 to 10 (0 indicating the worst and 10 the best) (see details in Table 1). Daily functioning in areas of work, social life and home was asked using a Finnish scale [23], adopted from Sheehan’s disability scale [24]. Participants rated their current work ability in comparison to their lifetime best [25], a reliable predictor of future work ability [26]. We also asked patients to assess their ability to work as fully, partially, or completely unable [27] with the idea of describing presenteeism/absenteeism due to PCC. Additionally, patients rated their current health and their experiences of being heard by health professionals and RTW negotiations, using the VAS from 0 to 10. In Finland, RTW negotiations involve employees, supervisors and occupational physicians. These meetings aim to temporarily reduce the current workload or modify work tasks, where possible, to support work ability and facilitate RTW [28].

Demographic and clinical data were collected electronically and with paper and online surveys. This information was merged with registry and clinical data from the HUS Data Lake on the HUS Academic platform. Comorbidity data from patients’ healthcare records covered the past five years of tertiary care and the HUS acute departments. Data from other primary or occupational healthcare sources was not included.

### Analytical approach and process

We used an inductive thematic approach, a flexible qualitative method suitable for analyzing diverse data types, particularly when prior studies are limited or fragmented [29]. The flexibility and reflexivity of this method allow researchers to clarify theoretical assumptions and justify choices made during the research process. This tends to yield a rich description of the entire dataset. We focused on the subjective meanings and perceptions that participants attributed to factors influencing work ability and the RTW process to gather insights specific to our study.

Three researchers (AS, AV, KK) conducted the analysis. AS is an MD and Occupational Health Specialist. AV is an MD, PhD and Occupational Health Specialist, while KK holds a PhD in Pharmacy and is a specialist pharmacist. Both AV and KK have prior experience in qualitative research. Collaboration occurred through online meetings, encouraging all researchers to share their views, reflect on the process and contribute to the discussion.

The analysis was carried out in several steps. The analytic process began by reading the transcriptions. First, relevant quotes were identified and given descriptive codes based on their meaning. Next, similar codes were grouped together into sub-themes. These sub-themes were then organized into broader upper themes. Finally, the upper themes were combined into main themes that summarized the key themes. The summary and themes are described in Appendix A. AS completed the initial coding and analysis. Each of the three authors (AS, AV, KK) read the interviews independently, and disagreements were resolved through discussion and consultation weekly. If necessary, we consulted the fourth reviewer (HL) and resolved disagreements through discussion and consensus. Through this process, we identified the RTW-related factors.

Descriptive statistical analysis of demographics and characteristics was performed using R version 4.2.3. Demographic parameters and the structured questions were computed and reported, with total and subdomain scores [frequency, mean, range, standard deviations (SD)].

## Results

Table 2 shows the demographics and characteristics of the interviewed participants (*n* = 28) and those considered for interview, including pilots (*n* = 67), with no significant differences between the groups at the first clinic visit. Interviewed participants had an average age of 46 years, and 71% were female. They were employed across various sectors, including education and childcare (*n* = 4), administrative occupations (*n* = 11), information technology (*n* = 4), construction (*n* = 1), logistics (*n* = 1), healthcare and social services (*n* = 5), cultural and leisure (*n* = 1) and finance (*n* = 1).

**Table 2.** Comparison of demographics and characteristics of analyzed patients and those considered for interview (including pilots) at the time of first clinic visit.

	Considered for interview, including pilots (*n* = 67)		Included in analysis (*n* = 28)		*p* Value^c^
Female, *n* (%)	51	(76.1)	20	(71.4)	.60
Age, years, mean (SD)	42.0	(8.8)	46.2	(9.2)	.10
Tertiary education, *n* (%) (*n* = 92)	41	(62.1)	14	(53.8)	.48
Social status^a^					
Full time employment, *n* (%)	38	(56.7)	19	(67.9)	.37
Part time employment, *n* (%)	8	(11.9)	6	(21.4)	.37
Student, *n* (%)	2	(3.0)	0	(0.0)	.56
Unemployed/job seeker, *n* (%)	3	(4.5)	0	(0.0)	.56
Retired, *n* (%)	1	(1.5)	0	(0.0)	>.99
Sickness allowance, *n* (%)	4	(6.0)	2	(7.1)	>.99
Rehabilitation subsidy, *n* (%)	13	(19.4)	3	(10.7)	.39
Other, *n* (%)	11	(16.4)	1	(3.6)	.10
Work ability assessment (*n* = 93)					.31
Fully able to work, *n* (%)	6	(9.1)	4	(14.8)	
Partially able to work, *n* (%)	46	(69.7)	15	(55.6)	
Completely unable to work, *n* (%)	14	(21.2)	8	(29.6)	
Comorbidities					
At least one comorbidity, *n* (%)	39	(58.2)	15	(53.6)	.82
Number of comorbid conditions, mean (SD)	1.3	(1.5)	0.8	(0.9)	.29
Hospitalization during acute COVID-19, *n* (%)	0	(0.0)	1	(3.6)	.31
Symptom duration at first clinic visit, years, mean (SD)	0.7	(0.5)	0.7	(0.4)	.30
Self-rated functional ability^b^, mean (SD)	4.9	(2.2)	4.7	(2.2)	.81
Self-rated quality of life^b^, mean (SD)	5.1	(2.0)	5.7	(2.4)	.18

*Note:* PCC: post-COVID-19 condition; n: numbers; SD: standard deviation; *p* value: probability value.

^a^ A patient may have chosen one or more options.

^b^ Self-reported on a scale of 0–10 (0 = poor; 10 = excellent) [18].

^c^ Chi-squared test for categorical variables, Mann-Whitney U for continuous variables.

At the time of the interview, the patients (*n* = 28) had been experiencing symptoms for an average of 14 months, ranging from 7 to 17 months. When assessing their work ability, 54% (*n* = 15) of patients considered themselves fully able to work, while 28% (*n* = 8) were partially able, and 18% (*n* = 5) were completely unable to work. Structured questions served as background information and discussion prompts, addressing health, work ability, daily functioning and experiences with health professionals. These aspects were self-rated on a scale of 0–10, where higher scores indicated better and lower scores indicated poorer functioning or condition. The mean self-assessed health score was 6.3 (range 2–9) and current work ability averaged 6.4 (range 0–10). In daily functioning, the work score averaged 6.4 (range 0–10), suggesting decreased occupational functioning. Social functioning also averaged 6.4 (range 2–10), while home functioning was higher, at 7.2 (range 2–10). Regarding experiences with health professionals, 64% (*n* = 18) rated being heard as good (scores 8–10), 11% (*n* = 3) as fair (scores 6–7) and 14% (*n* = 4) as poor (scores 0–5), with data unavailable for 11% (*n* = 3). RTW negotiations occurred in 43% (*n* = 12) of cases, with 75% (*n* = 9) finding them helpful.

Through qualitative analysis, we identified several factors affecting work ability and RTW, developing them into five themes: individual-related, work-related, health care-related, social environment-related and social insurance system-related factors (Table 3). The sub-themes within these groups and open coding are detailed in Appendix A.

**Table 3.** Summary of main themes about facilitators and barriers for return to work (RTW) and work ability.

Facilitators		Barriers
Ability for self-directed rehabilitation Stress and daily life management Motivation Ability to maintain positivity, patience and self-compassion	Individual-related factors	Severe symptoms Inability for self-guided rehabilitation Symptom-related worries, fears and negative thoughts Various stressors
Opportunity to modify job tasks and workload Employers’ activity and motivation in the RTW process Employer support and trust	Work-related factors	Lack of possibility to modify job tasks and workload Employers’ negative attitude Lack of flexibility Insufficient psychological support at work
Experience of psychological support in meetings with health professionals Appropriate explanation of symptoms, rehabilitation counseling Multidisciplinary approach Good accessibility and availability of services	Health care-related factors	Insufficient psychological support in meetings with health professionals Lack of understanding and knowledge of the PPC Poor accessibility and availability of services
Support and understanding from family and friends Peer support	Social environment-related factors	Lack of psychological support from family and friends Lack of understanding of symptoms that are not visible
Partial sick leave benefit	Social insurance-related factors	The eligibility criteria for social security benefits not met in RTW support measures

*Note:* PCC: post-COVID-19 condition.

### Individual-related factors

The patients described maintaining a positive attitude and mental well-being as vital during recovery. Embracing peaceful self-healing without pressure and letting go of constant achievement were important aspects. Practices like pausing, slowing down, exercising patience and engaging in self-reflection, along with finding joy in simple things and meaningful activities, supported their well-being. Believing in resilience, trusting the healing process, cultivating a sense of safety and understanding symptom causes were also key to recovery and RTW motivation. Table 4 provides examples of quotes regarding sub-themes of individual-related aspects that support rehabilitation and RTW.

**Table 4.** Examples of quotes illustrating the individual-related factors that supported rehabilitation and return-to-work.

Sub-theme	Examples of quotes
Maintaining a positive attitude and mental well-being	‘And then it was about setting your own mindset—having the patience to give yourself permission to heal in peace. I wish I had realized that a little over a year ago. But now I understand it’. (Female in forties, interview nr 5) ‘But I do know that I have really good resilience. I try to think about everything in a positive way and turn things around for the better, that’s how I am..’. (Female in fifties, interview nr 22)
Work-related motivation and commitment^a^	‘Probably just my own desire to stay at work’. (Female in forties, interview nr 13) ‘But then again, the work is meaningful and, in a way, has brought a new kind of enthusiasm. It has been nice to be at work’. (Female in thirties, interview nr 9) ‘It’s probably the strong work orientation. That unless it’s a life-threatening situation, you just have to keep going or stay away from work. But no, I still felt like I had to push through somehow’. (Female in forties, interview nr 4) ‘But the mindset and attitude of the employer and supervisor greatly affect how it feels to return and how it’s organized. That’s really important’. (Female in fifties, interview nr 20)
Sufficient understanding	‘I have to say, at the Long COVID clinic, I felt that the physiotherapist appointment was the most useful. The physiotherapist explained first what was going on, about the nervous system, how high the stress level is, and how they try to bring it down’. (Female in sixties, interview nr 8) ‘It’s about getting information. The fact that things were explained in ways that resonated with how I was feeling. And it helped me relate to my own condition better…’ (Male in forties, interview nr 6) ‘And then there was also the doctor’s visit, the first one, which helped me understand the symptoms and how they affect the autonomic nervous system and the nervous system in general. How the virus attacks. It helped me understand the symptoms’. (Female in fifties, interview nr 19)
Physical activities	‘Last summer, we did a longer canoe trip that included walking, paddling, and other activities […] and a month ago, I went on a longer skiing trip. When I can do them at my own pace—really slowly—they are incredibly beneficial’. (Male in forties, interview nr 27) ‘I learned something—I call it the “queen walk”—where I walked really slowly. And when others passed me, I thought of it like I was a queen gliding along, waving slowly to those passing by. Somehow, I learned to slow down and realized how refreshing that actually is. It was really essential’. (Female in sixties, interview nr 8) ‘Then it improved to the point where I was able to walk a bit again around December. Like… five- to ten-minute walks or something like that’. (Male in forties, interview nr 6) ‘So I feel that exercise played a really big role because it’s my only real way of managing stress’. (Female in forties, interview nr 15) ‘But I’ve also done things like going to the gym or go for a run in the morning before work. I try to be as active as possible. It’s really a source of strength for me—I draw energy from it to keep going’. (Female in forties, interview nr 4) ‘I’ve been doing some bouldering. It’s actually a pretty good sport because you can easily take breaks to rest. I meditate daily and find it quite useful. I go for a walk every morning and feel that it’s beneficial’. (Male in thirties, interview nr 12) ‘So, gradually longer walks all the time. The pace has probably stayed about the same, but I’ve been slowly increasing it bit by bit. And now, on a good day, I reach about 12,000 steps. The biggest healing factor is time—nothing else’. (Male in thirties, interview nr 21)
Stress management, relaxation and techniques	‘I do breathing exercises. I also go swimming and take ice baths or cold plunges. It has helped’. (Female in forties, interview nr 24) ‘Generally, relaxation. I also always try to go outside regularly to clear my mind. At least the relaxation techniques and these things helped’. (Female in twenties, interview nr 11) ‘Probably the best thing was the physiotherapy session I had at the clinic. All the breathing instructions and exercises I got were a really good quick fix’. (Female in thirties, interview nr 9) ‘And then I started now, maybe one month ago or something, to do a little bit yoga, like seven minutes a day, so this, you know, the small units. So, yeah. But always looking that my, yeah, pacing, that my heartrate doesn’t go too much up’. (Female in forties, interview nr 10)
Normal daily activities, planning, or enjoying simple things	‘Reading a book, lying down, doing very simple household chores, and taking naps, for example. And then trying to go to bed on time to get eight hours of sleep, with some quality restorative sleep in between’. (Male in thirties, interview nr 1) ‘I try to sleep when I feel sleepy. I try to live as regular a life as possible in a certain way. And I try to enjoy the little things. I also try to do yoga and meditate’. (Female in fifties, interview nr 20)
Rehabilitation programs (CBT-based net therapy and AIR program) for symptom management and recovery^b^	‘So now, this program that I started doing through HUS—I actually felt really good about myself for the first time in a long while earlier this year’. (Male in thirties, interview nr 12) ‘The online therapy from the clinic—that’s what I did. I participated in the ten-week program, as you probably know. It was absolutely, absolutely, absolutely amazing. I can’t praise it enough. It’s the best. I think all people with long-term illnesses should do something like that. It was really good and made me reflect on things’. (Female in forties, interview nr 2)
Medical treatment	‘I’m way better since I take this Montelukast, especially this Montelukast. Because it took away this inflammation, so I don’t have this fever anymore. And I don’t have this heart pain anymore’. (Female in forties, interview nr 10) ‘Yeah, I have a beta-blocker, and it works. It’s the only thing that works’. (Male in thirties, interview nr 21)

*Note:* CBT: cognitive-behavioral-therapy; AIR: amygdala-insula retraining program; HUS: Helsinki University Hospital.

^a^ Also included in the work-related factors.

^b^ Also included in the health care-related factors.

The patients also described gradually increasing activities, such as walking, cycling, swimming and gym exercises, as self-guided rehabilitation that facilitated recovery and RTW. Previous sports involvement and resuming hobbies also positively impacted their rehabilitation.

You shouldn’t expect too much from yourself or demand too much from yourself. It will come with time. I’ve also had a tendency to demand too much of myself, but now I’ve gradually come to understand that it’s not worth it, it doesn’t help anything. (Female in twenties interview nr 11)

In a way, being able to walk at least a little all the time, and then having new opportunities open up, like being able to swim and especially now being able to get around by bike, allows me to get a taste of more intense exercise. These are like echoes from the past, which is good when I get to return to them. (Male in forties, interview nr 7)

It’s a kind of gentle exercise, and then I also did some mindfulness, and I even asked if I could do it at the same time. They said yes, and that really helped too. (Female in forties, interview nr 2)

Stress management was supported by a diverse mix of coping strategies, including relaxation techniques, such as mindfulness, mental strategies and physical activities like yoga and cold-water swimming. These health behaviors, along with overload strategies like pacing, breaking down tasks and routines and ensuring adequate sleep, supported well-being and recovery. In addition to normal daily activities, simple pleasures, like crossword puzzles, caring for pets and enjoying nature or the sauna, contributed to stress relief. Medical treatment, such as beta-blockers helped manage palpitations.

. maybe I gave myself permission for restorative activities both during work and free time, and I did things that were good for myself, and that was a turning point in alleviating the symptoms. (Female in forties, interview nr 13)

We identified several barriers to RTW, primarily related to patients’ diverse symptoms, such as fatigue, pain, cognitive impairments and sleep problems. Palpitations and feelings of being overwhelmed hindered recovery and rejuvenation. Symptomatology led to a decline in functioning, making daily activities and even small exercises challenging.

One is the difficulty of concentrating. Sometimes it feels difficult to remember ordinary words or people’s names in meetings. So there’s a lot of forgetting in the middle of a sentence. (Male in forties, interview nr 27)

It somehow feels like the body is constantly in a state of alertness. That it’s like, it’s difficult to even try to calm down, so the body is somehow revved up. (Female in fifties, interview nr 3)

At first, it felt like I could manage a full workday, but pretty quickly, I realized I couldn’t. I simply didn’t have the energy, and I would completely crash before the workday was even over. (Female in forties, interview nr 25)

As emotional and psychological barriers, experiences of worry, catastrophic thinking and feelings of hopelessness and frustration regarding symptoms and recovery prognosis were common. Patients also reported demands and expectations about their capabilities and recovery. Those with comorbidities faced additional RTW challenges. Reduced stress tolerance and a tendency to push limits were obstacles to maintaining work ability.

The initial fear was also, first of all, whether I would ever be able to do these things again. (Female in sixties, interview nr 8)

I constantly have these big projects and change initiatives, so my memory and ability to absorb new information have declined. I constantly need to grasp new things very quickly, but my ability to do so has weakened. I also experience memory-related word and concept forgetfulness. [.] if I have to talk about names or say a specific term, I won’t remember it. Then I notice myself redoing tasks, or the information comes back to me later—it definitely has an impact. And since this has been going on for so long, I do feel a bit anxious about the whole situation. (Female in fifties, interview nr 22)

### Work-related factors

Adaptable work environments facilitated the RTW process through gradual part-time arrangements, such as partial sick leave (40–60% workload) or self-initiated workload reduction (80%). Occasional vacation days provided financial relief and substitute or replacement work supported a gradual transition to full-time RTW. Remote working reduced travel-related stress and allowed for necessary breaks, while in-person work remained important for social interactions. Table 5 presents examples of quotes related to sub-themes of work-related facilitators.

**Table 5.** Examples of quotes illustrating the workplace-related factors that supported rehabilitation and return-to-work.

Sub-theme	Examples of quotes
Flexible work arrangements	‘My work tasks have been adjusted according to my endurance. For example, I used to be responsible for leading meetings, but I found them stressful, so I stopped doing them, and someone else took over. I also had a lot of different things to take care of, so some of those responsibilities were reduced so that I could focus better on a few key tasks’. (Male in forties, interview nr 6) ‘The employer hasn’t set any kind of restrictions; they’ve been very flexible with this the whole time. Since I started working at 50% capacity, after a slight dip at the beginning, things have clearly improved over these two and a half months’. (Male in fifties, interview nr 26) ‘I worked part-time while receiving partial sickness allowance to make my return to work possible. The part-time arrangement and the employer’s support were crucial. Occupational health also provided support. And being able to return to my old job gradually, in small steps at first, really helped’. (Male in thirties, interview nr 1) ‘I can work at my own pace and have some influence on what tasks I do and when. Also, there’s flexibility in working hours—if I feel better one day, I can do a bit more, and if it’s a tough day, I can leave earlier’. (Male in forties, interview nr 7)
Remote work possibilities	‘Because I have the luck that I can work from home, so I can do that work. that is that I can work from home, so, that is supporting me that I have my silence and can concentrate on the things. That and, of course, also this pacing, so I have to take care of that I don’t get stressed or something, then I have to take distance and take breathing exercise’. (Female in forties, interview nr 10) ‘And luckily, since I work remotely from home, I’m able to take breaks and pace my work really well’. (Female in forties, interview nr 5)
Supportive work practices and work environment	‘But in that regard, my employer has really been supportive, giving me the space to recover at my own pace. For my recovery, having a good work community—a warm and caring environment—has been incredibly beneficial. Being able to laugh with colleagues instead of being sick alone at home makes a huge difference. I truly believe that a good work community is rehabilitative. It has brought me so much joy. It has helped tremendously that I have such supportive colleagues. I still have a yellow Post-It note stuck to my work screen that says, “You are a wonderful person, but your working hours are over”’. (Female in sixties, interview nr 8) ‘I have such wonderful colleagues and a great supervisor. The doctors and everyone here have been incredibly supportive, always encouraging me to rest and take it easy […]“So back then, my colleagues and I agreed that I would only do half a procedure day, so I didn’t have to work a full day”’. (Female in fifties, interview nr 19) ‘Yeah, this is a new, bigger project, which has actually been really exciting. Back then, that excitement really helped me return to work as well’. (Female in forties, interview nr 24) ‘My supervisor has been very supportive. They have said that there are no deadlines or anything, and that the most important thing right now is that I manage to stay at work somehow’. (Female in forties, interview nr 25)
Occupational health and employer collaboration (RTW negotiations)^a^	‘It was this kind of tripartite negotiation, where we were with the supervisor, the occupational health doctor, and me. We were just thinking about how the return to work should be structured, how to schedule it, what kind of tasks should be avoided, and what could be done. So, things like that. But in a way, it has worked as expected. The doctor provides support regarding what might be expected and how to approach things like an increase in workload’. (Male in forties, interview nr 7) ‘The doctor also talks with my supervisor while I am present. It helps immensely. So the fact that we are all present and agree on the ground rules together makes it so much easier compared to if I had to talk to the employer alone while being unwell’. (Female in sixties, interview nr 8)

*Note:* RTW: return to work.

^a^ Also included in the health system-related factors.

Facilitators included a variety of strategies, including modifying work content, adjusting tasks, reducing job responsibilities, managing work hours and overall workload and working at a comfortable pace. Breaks and task-sharing among coworkers supported work ability. Employers and colleagues who provided emotional support, encouragement, understanding, fairness, trust and recognition contributed to successful RTW. Also, meaningful aspects like having a sense of purpose, satisfaction, appreciation, autonomy and financial stability, which also relate to individual factors, acted as facilitators (see Table 4: Work-related motivation and commitment).

Yes, my own contribution has definitely been that I know which things burden me, and practical work planning arrangements, such as how I schedule meetings and other cognitively demanding tasks like job interviews, or alternatively, the possibility of remote work. These are the tools I have used, and they have practically been helpful to me. (Female in fifties, interview nr 23)

Workplace dynamics and challenges hindered the RTW process, including negative attitudes from employers, a lack of understanding of ‘invisible’ symptoms and reluctance or inability to provide support and trust. Additional stressors included work-related pressure, heavy workloads and limited task control. Obstacles also arose from demanding jobs, which contributed to cognitive and mental strain (an individual-level factor influenced by work conditions) as well as poor work atmosphere, staff turnover and shortages and constant changes in the work environment.

Work still causes mental strain. And I can’t handle it, my body can’t process it. So, if I work too much or too intensely, I just get exhausted. Even to the point where it continues into the next day and so on, so it just doesn’t work. (Female in forties, interview nr 5)

They (employer) want to consider transferring me to a completely different job and haven’t provided any support at all. I get the feeling that they might not even want me back. (Female in forties, interview nr 2)

The patients struggled to balance work and recovery, as their energy was consumed by their job, leaving no time or resources for other activities. Increased sensitivity to noise and difficulty with social interactions made being physically present at work challenging.

A lot of my energy goes into work, so in that sense, my social life has narrowed somewhat. After the workday, I just don’t have the energy to do much. My free time and evenings are partly spent recovering. (Female in fifties, interview nr 3)

I noticed that loud noise or situations where multiple people were talking at the same time, even in a break room, made me feel—maybe not a full panic attack, but something close to panic. I just couldn’t focus on anything, and it started to feel overwhelming. (Female in forties, interview nr 4)

### Health care-related factors

The patients described that in the OHS physicians facilitated RTW through various means, including providing medical statements, conducting examinations, making referrals and offering counseling. The OHS employed a multidisciplinary approach that offered mental health support, including short-term therapies, psychologist and physiotherapist sessions and specialist consultations. Professionals were praised for their active listening, understanding and realistic attitudes, which fostered patients to feel included. Key advantages were accessible appointments, regular monitoring of well-being and work ability and effective RTW organization.

RTW negotiations with employers were instrumental in clarifying task modification. Cooperation among healthcare providers and comprehensive service coverage were deemed essential.

But now, with regular meetings and the same doctor, it is now reassuring. (Female in fifties, interview nr 20)

A tripartite negotiation like this, where I was with my supervisor and the occupational health doctor. We were thinking about how to schedule the return to work, what kind of tasks should be avoided, and what could be done. So, things like that. But overall, it has worked as expected. (Male in forties, interview nr 7)

The patients praised the post-COVID-19 rehabilitation clinic for its comprehensive multidisciplinary approach, which provided readily available support. Communication with healthcare professionals was easy and secure, offering clear explanations and a deep understanding of symptoms. This alleviated patients’ anxieties and made them feel heard and understood.

Also, talking with you (clinic personnel) has provided me with mental support in this, that these symptoms are actually like this, that they are not solely due to myself. (Female in forties, interview nr 4)

Talking with all these experts really helped me understand my condition, my situation, and the future. […] It gave me a really positive and hopeful feeling—I wasn’t as worried about the situation anymore. Those conversations alone took a huge weight off my shoulders. (Male in forties, interview nr 27)

Experiences at the clinic showed the benefits of both individual and group counseling sessions by psychophysical physiotherapists for recovery. Patients valued the peer support and insights gained from body care group sessions focused on gentle movement, while individual sessions provided effective symptom management strategies. Additionally, CBT-based net therapy and the AIR program were instrumental in managing symptoms and aiding recovery (see also Table 4).

I was in a group where we did some light exercises at the beginning. And the exercises were so gentle, the kind that even elderly people do—it didn’t feel like much at all. But every time after the session, I felt more energetic, like my brain had been aired out. I felt refreshed, and even my eyes seemed brighter. If you’ve never experienced that physically, you just can’t believe it. It really made me realize what level of activity was right for me at that moment.[.] I never would have believed how much those peer groups helped and how far we got together. It was incredibly valuable to finally be around people who truly understood in their own bodies what we were talking about. That was a huge support, even though we didn’t meet many times. (Female in sixties, interview nr 8)

The online therapy from the clinic—that’s what I did. I participated in the ten-week program, as you probably know. It was absolutely, absolutely, absolutely amazing. I can’t praise it enough. It’s the best. I think all people with long-term illnesses should do something like that. It was really good and made me reflect on things. (Female in forties, interview nr 2)

Barriers related to healthcare included a lack of emotional support, temporary solutions, limited service availability, poor communication among providers and insufficient understanding of PCC. Some patients found interventions like net-therapy and the AIR program cognitively taxing. RTW negotiations felt futile or cumbersome, for example, when rehabilitation plans did not meet patients’ expectations or agreed-upon modifications were not implemented at work. A key issue was differing priorities: while physicians focused on RTW, patients prioritized their well-being and coping abilities.

I visited occupational health services, and the understanding there was really weak. The doctor admitted upon my arrival that they were not familiar with the issue and did not know what mechanisms could be used to support my situation. (Female in fifties, interview nr 23)

It was quite superficial and quick handling. They didn’t delve into anything but always tried to refer to someone else, so it left a taste in the mouth that they just wanted to give some temporary solution and then wait for a while to assess the situation again. (Male in thirties, interview nr 1)

### Social environment-related factors

As a cross-cutting element, social perspectives emerged as central across all themes. Psychological support and emotional safety were crucial for RTW. The patients found such support indispensable not only in work and healthcare settings but also within family context. They benefited from the understanding and encouragement of family, friends, employers and colleagues. Additionally, peer support helped them realize they were not alone in facing their challenges.

We talked in that group, and in the end, we all had some previous experience that led to how much it’s about feeling safe. In that group, you feel safe. And everyone has their own story. (Female in sixties, interview nr 8)

We are a family of five here with three teenagers, so that has actually been quite beneficial. The children are at an age where they help with household chores, take care of shoveling snow, and various other things. (Male in forties, interview nr 7)

Conversely, the absence of psychological support hindered progress, leaving some patients feeling marginalized, particularly when symptoms were ‘invisible’.

But then if you have a broken leg, people understand that you can’t walk. You’re just like all your muscles weigh ten tons and you just can’t. So, it’s really difficult to try to explain that to anyone on the outside. (Female in forties, interview nr 5)

### Social insurance-related factors

Partial RTW was arranged by the Social Insurance Institution through partial sick leave when benefit criteria were met, enabling many to RTW.

Indeed, I worked part-time on a partial sick leave allowance to make this return to work possible. (Male in thirties, interview nr 1)

However, obstacles arose when patients did not meet the criteria for receiving benefits. This led to feelings of injustice and frustration, as the process was perceived as complicated and disappointing. Patients frequently had to advocate for their rights, as the insurance system often failed to recognize their symptoms as legitimate illnesses, adding to their burden.

I applied to the pension insurance company, under the name of vocational rehabilitation, and received a rejection because the reason was that there is no threat of complete loss of work ability. (Male in forties, interview nr 7)

No, the pension insurance company does not see this as an illness at all; they do not in any way acknowledge the matter. (Male in forties, interview nr 18)

## Discussion

### Main findings

This study explored the perceptions of patients with PCC regarding how the condition impacts their work ability and RTW, aiming to deepen understanding of patients’ needs and the complexities involved in the RTW process. Our findings highlight the intricate and multifaceted support required for patients with PCC, with individual experiences and social aspects playing a central role. The study underscores the critical need for patients to feel seen, understood and supported by various stakeholders, including employers, family, healthcare professionals and compensation system. Social support emerged as a pervasive element across different perspectives and areas of recovery, emphasizing the importance of psychological support. Additionally, the active roles, mindsets, emotions and motivations of individuals were key contributors to the rehabilitation and RTW process.

### Complexity of work ability and RTW process

Through our inductive analysis, we developed five themes affecting work ability and RTW, each encompassing facilitating and hindering factors: individual-related, work-related, health care-related, social environmental-related and social insurance system-related factors. We observed similarities between these themes and Loisel’s arena model for work disability prevention [30], which connects RTW determinants not only to patients’ physical and psychosocial characteristics but also to various stakeholders, such as the workplace, healthcare system and compensation system. Our findings are consistent with the model’s elements that address cooperation challenges within this multi-player system, which is essential for preventing disability [30]. In our study, social perspectives emerged as a cross-cutting element across all four other themes, forming a distinct theme on its own. In Loisel’s model, the different levels, including the personal element, emphasize a work rehabilitation approach that considers the worker’s physical, cognitive and affective characteristics and social relationships. RTW opportunities include rehabilitation services provided by various management structures, whether independent or multidisciplinary. In addition, workplace facilitators and barriers vary from job specifics to broader external factors, and there is interaction between the worker and the compensation system involved [30]. Notably, in our study, many facilitators and barriers to recovery and work ability support were more related to psychosocial, workplace and management issues than to PCC itself.

### Comparison with existing literature

As background information and discussion prompts, we also used structured self-rated measures of work ability and everyday functioning. Although, at the time of the interview, the majority (82%) perceived themselves as fully or partially able to work, they still rated their work ability as reduced (6.4 out of 10) compared to their lifetime best. This finding of reduced work ability aligns with previous research indicating that many patients with PCC continue to face work ability challenges upon RTW [9,10]. Moreover, our study observed functional impairments were more broadly in daily life activities and participation, particularly in social interactions and, to a lesser extent, in daily home activities.

The WHO advocates for a multi-professional approach to rehabilitation for patients with PCC [31]. Our findings support this recommendation, emphasizing the need for healthcare practitioners to extend their focus beyond medical care and address broader factors, thereby enhancing rehabilitation, supporting work participation and facilitating RTW. Positive experiences from RTW negotiation – serving as a cooperative forum between OHS and the workplace – highlight the importance of collaboration in facilitating a successful RTW process [32]. In this multifaceted approach, our patients described the crucial role of employers in creating a supportive and accepting work environment. By recognizing and valuing the efforts of employees facing health challenges, organizations can strengthen their sense of belonging and commitment, fostering a positive organizational culture that aids recovery and the RTW process [32,33].

However, our patients often reported their suffering was not taken seriously or recognized by healthcare providers, family, friends and within the workplace. This reflect a typical experience for those with PPS [34]. Our findings of skepticism and lack of understanding about PCC in workplaces and among healthcare providers align with existing research, which has shown these attitudes can hinder rehabilitation and the RTW process [11]. Additionally, our study revealed issues with the social benefits system, which did not always acknowledge patients with PCC, leading to struggles in qualifying for financial support for partial RTW benefits or sick leave.

Patient active participation in rehabilitation and RTW aligns with previous research. This involvement fosters responsibility, resilience and motivation, resulting in better outcomes [35].

Our study identified symptomatology, particularly fatigue and cognitive issues [8], as major obstacle for RTW. These findings resonate with evidence not only for patients with PCC but also for those with PPS and other chronic diseases, such as chronic pain, mental disorders and neurological diseases [35–37]. Patients with persistent health complaints often experience isolation, anxiety about relapses, uncertainty about recovery, fearing failure at work or disappointing employers and colleagues, which can lead to feelings of shame and perceived burden. This can exacerbate suffering. In addition, research on PPS highlights the need to prevent symptom avoidance in rehabilitation [37]. Our findings indicate that patients with PCC also experienced fear and engaged in avoidance behaviors due to symptoms, which can worsen their condition by limiting engagement in normal activities. This can create a harmful cycle that hinders RTW [36].

Patients in our study often reported psychosomatic physiotherapy – which addresses both physical symptoms and their psychological components – and CBT-based internet therapy [22] as helpful for managing symptoms and stress. However, due to the study design, definitive conclusions about their effectiveness cannot be drawn. Previous evidence on CBT-based therapies and physical exercise as rehabilitation tools for PCC shows improvement in quality of life, fatigue and concentration symptoms. Nonetheless, the evidence remains limited and conflicting, possibly because patients with PCC are heterogeneous, with symptom severity and fluctuation affecting study outcomes. Further research is needed to assess rehabilitation interventions for PCC.

Our research amplifies the patient’s voice and experience through qualitative research methods, providing insights from non-hospitalized patients and enhancing existing knowledge about their challenges and needs.

### Implications for staying at work

Given the multifactorial nature of PCC, our study highlights the necessity of a holistic approach that incorporates individual, social and work-related factors to support RTW effectively. Our findings underscore the role of healthcare providers and occupational health practitioners in delivering tailored care and support through a multidisciplinary approach that encourages patient participation. This includes user-centered interventions focused on daily activity, gradual exposure to work tasks [33] and coping strategies to manage symptoms and psychosocial load. Recognizing underlying health conditions and psychosocial burdens that affect daily functioning and well-being allows for customized interventions that address disability, enhance recovery and promote RTW, benefiting both patients and employers. Effective RTW support requires collaboration among healthcare providers, employers and the social security system, especially in prolonged cases.

In a multifaceted approach, it is crucial to go beyond adjusting tasks and workloads [9,12] by ensuring employees feel safe discussing their limitations and work arrangements [32,34], as this can enhance their willingness to RTW. Emotional and psychological support from family and friends forms a vital component of the patients’ supportive social network [36]. Addressing economic insecurity and recognition issues within social benefit systems is important to alleviate stress and anxiety among patients.

In healthcare, actively engaging patients in discussions about their fears, coupled with education to reframe beliefs and encouragement to take an active role and to engage in physical activity, supports rehabilitation progress [36]. Physical activity is recommended for most medical conditions, as it improves overall health, quality of life and recovery, including for patients with PCC [38]. Clinicians can support patients by raising awareness of the benefits of physical activity and tailoring advice to accommodate relapses and symptom variability. Encouraging self-directed rehabilitation can increase adherence to treatment plans and help patient to set achievable goals. A new chronic condition can naturally evoke negative emotions, thoughts and fears about potential loss of health and functional ability. Clinicians can address these emotions by providing compassionate support and opportunities for open discussion to foster hope for recovery.

### Strengths and limitations

A key strength of this study is its in-depth exploration of patients’ perceptions and experiences with RTW and rehabilitation. The diverse age groups and occupations of the patients provided a broad perspective. All patients had a confirmed COVID-19 diagnosis *via* PCR testing and were treated at an outpatient clinic for PCC, ensuring other medical conditions were ruled out. Interviews were transcribed verbatim, minimizing researcher bias by not relying on notes and memory. The majority of patients were not hospitalized for their acute COVID-19 infection, enhancing the representativeness of our sample to reflect the general PCC population. This contrasts with many qualitative studies relying on online surveys [11,12] or specific occupational groups [12], which may not reflect the broader PCC population. Characteristics for the participants (*n* = 28) and those considered for interview (*n* = 67), including pilots, showed no significant differences between the groups at the first clinic visit, enhancing transferability. The pilot interviews were also a strength, as they verified the semi-structured interview technique, ensuring effective facilitation of patient-centered dialogue without requiring modifications to the interview guide. Data saturation was achieved as similar factors affecting work ability were consistently discussed, underscoring the robustness of the findings and the sufficiency of the sample size.

This study has limitations. As a cross-sectional qualitative study, it lacks follow-up, which limits insight into long-term RTW outcomes. Our findings apply only to working-aged patients who were non-hospitalized during acute COVID-19, were employed or had plans to RTW. In addition, the overrepresentation of female participants (over 70%) limits transferability. The research focused on events and experiences from the previous period related to work ability and RTW. There is potential selection bias since participation was based on voluntary enrollment and the sample consisted of clinic patients who may be more receptive to rehabilitation and psychoeducation. Recruitment was swift due to high motivation, as many participants considered the study important for highlighting work ability with PCC. While patient subjectivity is valuable for understanding experiences, memory bias and social-desirability bias could influence the results. The research sample was mainly from ‘white collar’ professions, with distinct physical requirements compared to other groups. The researcher’s background in occupational health might have shaped the study design; however, subjectivity was minimized through the broad experience and active involvement of all research team members in the design and analysis process. To address potential limitations in inter-rater reliability, the coding process was conducted collaboratively within the research team, with discussions to ensure consistency and consensus. The daily functioning measure was adapted from the Sheehan Disability Scale and is not validated, though it showed similar patterns to the work ability measure. Despite variations in healthcare and compensation systems, patient perspectives on work ability and RTW are applicable in diverse contexts and likely relevant beyond the Finnish context, though caution is advised in generalizing results.

## Conclusion

PCC is inherently multifactorial, with complexity further compounded by challenges in work ability and the support required for RTW. This multifaceted nature necessitates a holistic approach, considering individual, social and work-related factors. Effective support involves understanding patients’ experiences and fostering collaboration among healthcare providers, employers and the social security system to facilitate RTW, particularly in prolonged cases.

## Appendix A.

**Table A1.** The summary of the categories of the inductive content analysis.

Main category	Upper category	Sub-category	Open coding
Facilitators	Individual-related factors	Maintaining a positive attitude and mental well-being	The importance of maintaining a positive attitude and motivation during recovery.
			Believing in resilience and trusting the healing process, embracing peaceful self-healing without pressure
			Practicing self-reflection, patience, and examining one’s emotions.
			The importance of slowing down and pausing.
			Finding joy in small moments and engaging in meaningful activities.
		Sufficient understanding	Nurturing a sense of safety.
			Understanding the causes of symptoms as a support for recovery.
			Maintaining motivation for returning to work
			Accepting the recovery process as part of daily life.
		Gradually increased activities	Walking, cycling, swimming, and gym exercises as self-guided rehabilitation
			Previous sport involvement
		Stress-management and relaxation techniques	Practices like mindfulness, relaxation, and mental strategies were key for stress management.
			Activities such as yoga and cold-water swimming supported both well-being and recovery.
		Systematic routines and planning	Techniques like pacing, systematic planning, breaking down tasks and routines
			Ensuring adequate sleep was effective in managing stress and improving recovery.
		Enjoying of simple things	Engaging in activities like crossword puzzles, caring for pets, enjoying nature and sauna
		Medical support	Beta-blockers helped manage symptoms like palpitations
			The nutritional supplement has helped
			Paxlovid takes away the symptoms
	Work-related factor	Flexible work arrangements	Gradual work adjustments: partial sick leave, self-initiated workload, gradual transitions to full-time work, substitute tasks, occasional vacation
		Remote work possibilities	Remote work reduced travel-related stress, allowed for breaks and enabled better self-management of energy levels
		Supportive work practices and work environment	Modifying tasks, reducing job responsibilities, managing workloads
			Supportive coworkers and employers who fostered psychological safety, fairness, trust and recognition
			having meaningful tasks, a sense of purpose, satisfaction, appreciation, autonomy and financial stability
			scheduling cognitively demanding tasks and remote work
	Health system -related factors	The occupational health system (OHS)	Multidisciplinary approach
			Medical statements, examinations, referrals and counseling, along with mental health support such as short-term therapies, psychologist and physiotherapist sessions and specialist consultations
			Professionals active listening, understanding and realistic attitudes, fostering a sense of inclusion
			Accessible appointments, regular monitoring and structured RTW organization
			Joint discussions (RTW negotiations) between patients, employers and OHS professionals clarified task modifications and work arrangements, easing the RTW process
			Regular meetings with the same physician provided reassurance and continuity
		Post-COVID-19 rehabilitation clinic as a resource	Clinic offered individualized and group-based counseling, physiotherapy and medical expertise, particularly in addressing symptoms related to the autonomic nervous system
			Group sessions focused on gentle movement and body awareness provided valuable peer support and recovery insights
			Programs like CBT-based net therapy and the AIR program were instrumental for symptom management and recovery, despite some patients finding them cognitively demanding.
			The healthcare professionals’ clear explanations, empathy and understanding, which alleviated anxiety and reinforced a sense of being heard.
	Social environment-related factors	Psychological support	Understanding and assistance from family members, such as teenagers helping with household chores
			Encouragement and understanding in the workplace
			Engaging in peer support groups provided a sense of safety and reassurance. Sharing stories realizing they were not alone helped them process their experiences and build resilience
		Emotional safety	Peer groups highlighted as critical spaces where patients felt safe to share their experiences and build emotional resilience.
			Psychological support from family, friends, colleagues and healthcare provider helped foster emotional safety
	Social insurance -related factors	Partial RTW	The Social Insurance Institution’s partial sick leave benefits supported in arranging partial RTW
Barriers	Individual-related factors	Symptom-related barriers	Diverse symptoms such as fatigue, pain, cognitive impairments and sleep problems hindered RTW.
			Palpitations and feelings of being overwhelmed affected recovery and energy levels.
			Decline in functioning with even small tasks, like emptying a dishwasher, causing extended exhaustion.
		Emotional and psychological barriers	Experiences of worry, catastrophic thinking and feelings of hopelessness and frustration about symptoms and recovery prognosis
			Fear about whether they would ever regain their previous abilities
		Stress tolerance and behavior patterns	Reduced stress tolerance made it difficult to maintain work ability
			A tendency to push personal limits further complicated recovery efforts
		External demands and comorbidities	Patients faced external demands and expectations regarding their recovery and capabilities
			Comorbidities added complexity and additional challenges to the RTW process
	Work-related factors	Negative attitudes and work condition	Negative attitudes from employers, limited understanding of ‘invisible’ symptoms and a lack of trust or willingness to accommodate
			Heavy workloads, high job demands, limited control over tasks and poor work atmospheres (e.g. staff turnover, shortages and frequent changes)
			Feeling undervalued and seen as burdens rather than contributors by some employers
		Work-life balance challenges	Work overload depleting their energy and leaving no resources for other aspects of life
			Noise sensitivity, social interaction difficulties and mental strain from demanding tasks
			Overworking leading to exhaustion that persisted for days, making it difficult to sustain a productive work routine
		Employer attitude and relationship	Unprofessional or dismissive behavior
	Health system-related factors	Healthcare and RTW process	Insufficient emotional support and a lack of individualized care in rehabilitation planning.
			Interventions often felt short-term or poorly tailored, leaving patients feeling unsupported in the long term
			Poor coordination among healthcare providers and restricted access to services
			Counseling felt unrealistic or unhelpful when agreed-upon modifications were not implemented
			Misaligned priorities between patients and physicians: patients focused on well-being, while physicians prioritized RTW
		Patients’ expectations for recovery and rehabilitation	The need for personalized rehabilitation plans that align with their priorities, such as regaining physical function or managing specific symptoms.
			Patients wanted deeper, more thorough assessments and long-term solutions rather than referrals or quick fixes
	Social environment-related factors	Lack of psychological support	A lack of psychological support, particularly when symptoms were ‘invisible,’ left patients feeling marginalized and misunderstood
			Struggling to explain their condition to others, as symptoms like fatigue and heaviness were not easily visible or relatable compared to physical injuries
	Social insurance-related factors	Partial RTW	Faced barriers when the criteria did not meet for receiving partial sick leave or vocational rehabilitation benefits despite symptom
			Rejections from insurance providers led to frustration, disappointment and a sense of injustice.
			The process of applying for benefits was perceived as complicated and burdensome, forcing patients to advocate for their rights
		Lack of recognition for symptoms	The insurance system did not adequately recognize symptoms (e.g. long COVID) as legitimate illnesses, further adding to emotional and mental strain.
